# Bringing AI to the clinic: blueprint for a vendor-neutral AI deployment infrastructure

**DOI:** 10.1186/s13244-020-00931-1

**Published:** 2021-02-02

**Authors:** Tim Leiner, Edwin Bennink, Christian P. Mol, Hugo J. Kuijf, Wouter B. Veldhuis

**Affiliations:** 1 Department of Radiology | E.01.132, Utrecht University Medical Center, Heidelberglaan 100, 3584CX Utrecht, The Netherlands; 2 Image Sciences Institute, Utrecht University Medical Center, Utrecht, The Netherlands

**Keywords:** Artificial intelligence, Imaging informatics, Deployment, Workflow, Vendor-neutral

## Abstract

AI provides tremendous opportunities for improving patient care, but at present there is little evidence of real-world uptake. An important barrier is the lack of well-designed, vendor-neutral and future-proof infrastructures for deployment. Because current AI algorithms are very narrow in scope, it is expected that a typical hospital will deploy many algorithms concurrently. Managing stand-alone point solutions for all of these algorithms will be unmanageable. A solution to this problem is a dedicated platform for deployment of AI. Here we describe a blueprint for such a platform and the high-level design and implementation considerations of such a system that can be used clinically as well as for research and development. Close collaboration between radiologists, data scientists, software developers and experts in hospital IT as well as involvement of patients is crucial in order to successfully bring AI to the clinic.

## Key Points

- Production of artificial intelligence algorithms relevant for radiologists is ramping up.
- Uptake of artificial intelligence in everyday clinical practice is lacking.
- A dedicated vendor-neutral AI deployment infrastructure can help to overcome this barrier.
- A description of the components of a vendor-neutral AI deployment infrastructure is provided.
- Bringing artificial intelligence to the clinic requires a multidisciplinary team.

## Introduction: clinical uptake of AI is lacking

Artificial intelligence (AI), broadly defined as algorithms that can perform tasks that would require intelligence if performed by humans [1], is making increasing inroads in medical imaging. The ubiquitous availability of digital imaging data, cheap storage and graphics processing units (GPU) as well software to create AI algorithms have led to an exponential increase in algorithm development. Although AI is most often associated with image analysis tasks such as segmentation or detection, it is being applied much broader. For example, most major imaging equipment vendors are now introducing AI algorithms for image reconstruction on CT and MRI machines that enable faster reconstructions with less image noise. On the other end of the spectrum, AI techniques are used to extract prognostic information from diagnostic studies. For a review of basic AI concepts relevant for radiologists we refer to the European Society of Radiology (ESR) white paper [2]. For a discussion of the basic steps in the deep learning workflow in radiology we refer to the primer by Montagnon et al. [3].

Given these developments, the past few years have seen a huge surge of interest in development of AI relevant to practising radiologists. All around the world, researchers are creating algorithms that could solve a variety of problems along the continuum of radiological care, from triaging of patients for imaging, to image acquisition and reconstruction, reporting and extraction of prognostic information from diagnostic studies. In addition, one of the benefits to be gained by using AI is to reduce variability in reporting between different radiologists by automating tedious and repetitive steps and making them more quantitative and more precise. A PubMed search showed that the year over year growth in publication of new algorithms is nearly exponential; up until 2010, less than 50 papers were published in the medical literature using the search terms ‘machine learning’, ‘deep learning’ and ‘radiology’. At the time of writing, close to 4750 papers were published [4], which equals an annual compound growth rate of approximately 57%. Yet, there is very little evidence that these algorithms are actually used in clinical practice. In other words, deployment of AI algorithms in actual clinical practice is not commensurate with the rapid pace of development and production and there is a near total lack of evidence of patient benefit.

What are the reasons for this discrepancy? Is uptake delayed by time to obtain regulatory approval? This is unlikely because even when only looking at FDA and EMA-approved algorithms, there is little real-world uptake. Is the reason budgetary lag? Radiology departments will need to make funds available to purchase AI solutions and many are only starting to do so now. But given that AI is potentially cost-saving and that software is a low-single-digit fraction of the total radiological budget, this too cannot be the leading impediment to uptake. Is it the lack of strong evidence of clinically relevant improvements? This is a catch-22 since evidence on increased radiologist productivity and especially improvement in patient outcomes, will be hard to obtain without first implementing AI.

While all these factors need to be addressed, we believe the main impediment to widespread uptake of AI—and the solution—lies in solving the problem of *how* to actually deploy AI in clinical practice.

## Rationale for development of a vendor-neutral AI deployment infrastructure (VNAI)

The present model for procurement and deployment of computer-aided diagnosis (CAD) software—we will refer to this as ‘point solutions’ in the remainder of the article—does not apply to AI very well. In the near future, it is likely that there will be dozens if not hundreds of clinically useful AI algorithms with regulatory approval. This is not simply a luxury of choice. It is important to realize that for years to come these AI products will each individually address a narrow, very specific area of the total radiological workflow. Having dozens or more AI applications installed and working in concert, is a *conditio sine qua non* for crossing the threshold to demonstrate patient benefit. However, deploying and maintaining dozens or more individual (virtual) workstations or even software packages for all of these will be unmanageable.

Several vendors have attempted to address this problem by creating AI algorithm ‘marketplaces’, a concept similar to e.g. Apple, Inc’s App Store. Although this may seem a good solution at a first glance, it solves only part of the problem. While it may make procurement easier, it does not address actual clinical deployment. Furthermore, these marketplaces each individually will offer a fraction of all available algorithms. This not only limits choice but introduces the risk of vendor-lock-in, which is not desirable from a departmental, radiologist or patient perspective, especially when the marketplace is coupled to some sort of deployment pathway. To solve both the problem of deployment and of vendor-lock-in, over the past four years, the Department of Radiology at Utrecht University Medical Center has been developing a *vendor-neutral AI infrastructure (VNAI).*

The purpose of this article is to discuss the rationale for investing in a VNAI and to share our blueprint for such an AI deployment platform. We then discuss how results are presented to radiologists as well as governance and quality control. We end with a brief discussion of the ethical issues and share future perspectives including the potential of the platform for serving as the hospital’s central ‘AI-hub’.

## Rationale for investing in a VNAI

There are several reasons that make it worth investing in a VNAI. First, it provides a scalable architecture to easily deploy a large number of FDA and EMA-approved algorithms from different vendors in clinical practice. The platform can be set up to automatically run an algorithm based on specific programmable triggers. Also, cascades of algorithms that sequentially perform multiple different operations on data from the same patient can be preprogrammed. A second reason a VNAI is valuable is the ability to test and validate new algorithms prior to actually purchasing them. Investing in a VNAI is also beneficial from the perspective of both academic and commercial AI development because of the ability to test and validate research algorithms on real-world clinical data. Early testing and rapid feedback from clinicians create an iterative cycle of continuous improvement and provide important feedback about an algorithms’ accuracy and user-friendliness. In addition, being able to run algorithms locally also removes some of the substantial barriers to multi-institutional and multi-national collaborations by preventing the need for transferring patient data, which is often hampered by privacy regulations and concerns about anonymization. Another reason from a research perspective is that a VNAI platform can also be used for large-scale data labelling by multiple experts simultaneously. Radiologists can use the system to rapidly label images with specific keywords, annotations or other ground truth data that can subsequently be used to develop a new algorithm, even while performing clinical tasks.

IMAGR, the VNAI created at UMC Utrecht, was created with these requirements in mind and is a joint effort of the Department of Radiology, the Image Sciences Institute and the Advanced Data Analytics in Medicine program of the Utrecht University Medical Center. In the following sections we discuss the components of IMAGR, how users interact with the system and considerations regarding quality control, ethical aspects as well as future developments.

## The components of a VNAI

**Fig. 1:**
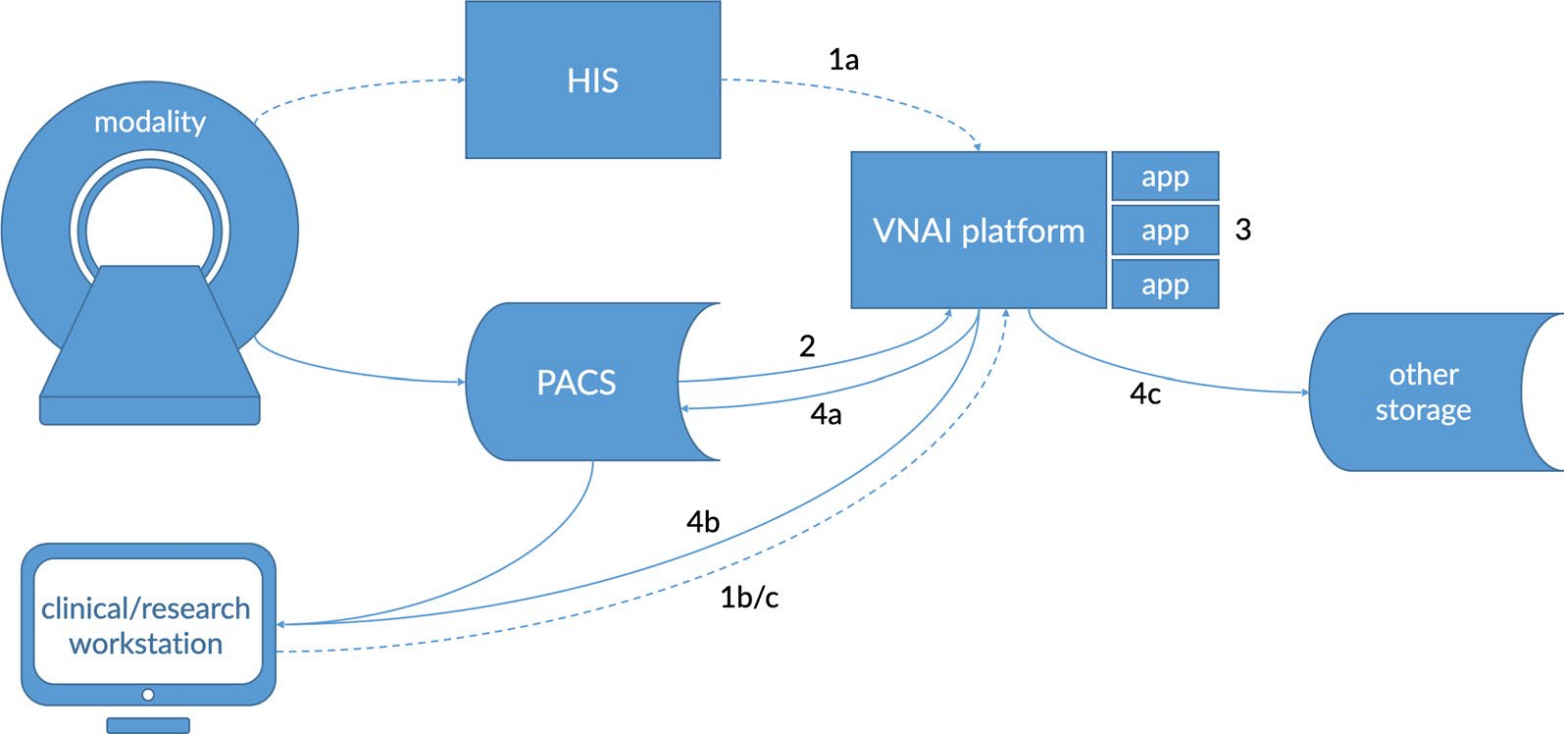
Interaction with the VNAI platform. Upon completion of image acquisition, the VNAI platform is notified through the hospital information system (HIS) (1a). This, in turn, triggers the VNAI to retrieve relevant images from the PACS system (2) and to run analysis algorithms (3). Subsequently, results are sent back to the PACS system (4a), are available through a dedicated viewing solution (4b) or sent to other forms of storage (4c). The VNAI can also be activated directly by clinical users (1b) or researchers (1c). Numbers refer to the outline of VNAI components in the text

One of the main tasks of a VNAI is the automatic application of image processing and image analysis to radiologic examinations. From a high level, there are four sequential key events that describe the essence of such a system (Fig. 1):

1. The system is triggered, for example:
  a automatically when image acquisition has finished
  b by a radiologist or technologist during reading
  c by a (bulk-)query from a researcher
2. Relevant image series are being retrieved from the PACS.
3. An algorithm pipeline is applied.
4. Results are made available, for example:
  a sent back to the PACS, whether as images, prioritization signal, etc.
  b through a dedicated viewing solution
  c to other storage, a database, email notification, etc.

In the next section, high-level design choices that were made for the platform components that execute these events are explained. There are, however, two aspects that all components have in common. In order to integrate the VNAI with the existing hospital IT infrastructure it is important to (1) adhere to industry standards for information exchange and to (2) build upon mature, proven technology whenever possible.

### Connection with the hospital information system

When image acquisition in a radiology examination is finished, the resulting images are sent to the picture archiving and communication system (PACS) and the hospital information system (HIS) is notified that this subtask has been completed. This notification triggers the PACS to put a new subtask—to examine the acquisitions—in the radiologists’ worklists. This notification is also processed by the VNAI, telling it that all images related to that specific examination are now available for AI processing as well.

Health Level 7 (HL7) is the most widely implemented messaging standard for communicating these notifications [5]. IMAGR was therefore designed to be subscribed to a HL7 channel that sends these messages and to take action when it learns that image data have become available that might be suited for automated analysis.

### Retrieving information and image data from the PACS

As HL7 messages do not contain any specifics on image acquisition, it is often necessary to query the PACS for additional information in order to verify whether the image data are suited for automated analysis. In any radiology PACS, Digital Imaging and Communications in Medicine (DICOM) is the standard for image storage, exchange and display. AI algorithms in IMAGR are embedded in so-called 'pipelines', a modular sequence of steps to retrieve, prepare and analyse image data and to process the results. Pipelines, including subsequent rules and filters, can be triggered by any set of DICOM tags. In case a study matches the pre-specified criteria for any pipeline, its relevant image series will be retrieved from the PACS and made available to all matching pipelines.

An example of such a set of criteria for a pipeline that provides brain volumetry from a three-dimensional T1-weighted MRI sequence is: [‘Modality’ equals ‘MR’, ‘Body Part Examined’ equals ‘BRAIN’,’ Patient's Age’ equal to or larger than 18, ‘Series Description’ contains ‘3D’ and ‘T1′]. When it comes to parsing these DICOM tags, it must be noted that standardization is key. Radiology technologists should therefore become ‘AI-aware’, i.e. they should keep in mind that information that they enter will be processed automatically. It is important to further note that there will still be tasks that require human input. The human intervention could be supplemented by dedicated algorithms performing automated data curation, prior to passing data on to an AI pipeline.

### Running algorithms

Being a platform that has to be able to run a wide variety of algorithms, each having different requirements and dependencies, containerization is an important underlying technology in IMAGR [6]. Containerization involves bundling an application together with all of its related configuration files, libraries and dependencies required for it to run in an efficient and conflict-free way across different computing environments. Each pipeline consists of several steps, all incorporated as a micro-service, running in its own isolated virtual environment that can be started and stopped on demand. By leveraging existing container software, resources can be shared, and a high level of security can be achieved. Storage and other expensive hardware, such as GPUs, become a shared resource that benefits all algorithms, resulting in higher utilization and better cost efficiency.

Not only the algorithms, but also the platform itself has been designed in a modular, containerized fashion, which has significant benefits. Containers, and therefore (parts of) the platform, can be easily moved to other hardware. Scaling should also prove to be easier when non-specific and thus cost-efficient hardware can be used. The isolated components can be updated without affecting the host system and other components, as they are minimally dependent on the host operating system. By fine tuning each component’s input and output profile, a high level of isolation can be achieved.

### Pipelines, modular execution and scheduling

Most image analysis algorithms can be split up in subtasks, or modules, of which many have generic functionality. This functionality can be e.g. file-format conversion or image pre-processing such as alignment, noise reduction or skull stripping, to name but a few. As mentioned earlier, the IMAGR platform defines AI algorithms as a concatenation, or pipeline, of such modules. This not only benefits algorithm development, but it also reduces computational load because subtasks that are common to multiple pipelines have to be executed just once. Another major benefit of this approach is the modules can be used as building blocks to create a new pipeline with the combined capabilities of its components. As a real-world example, an algorithm that detects white matter lesions on a whole brain MRI can be combined with another completely different algorithm that can segment a brain MRI into anatomic subparts, into a new pipeline that can quantify white matter disease per anatomic brain region.

**Fig. 2:**
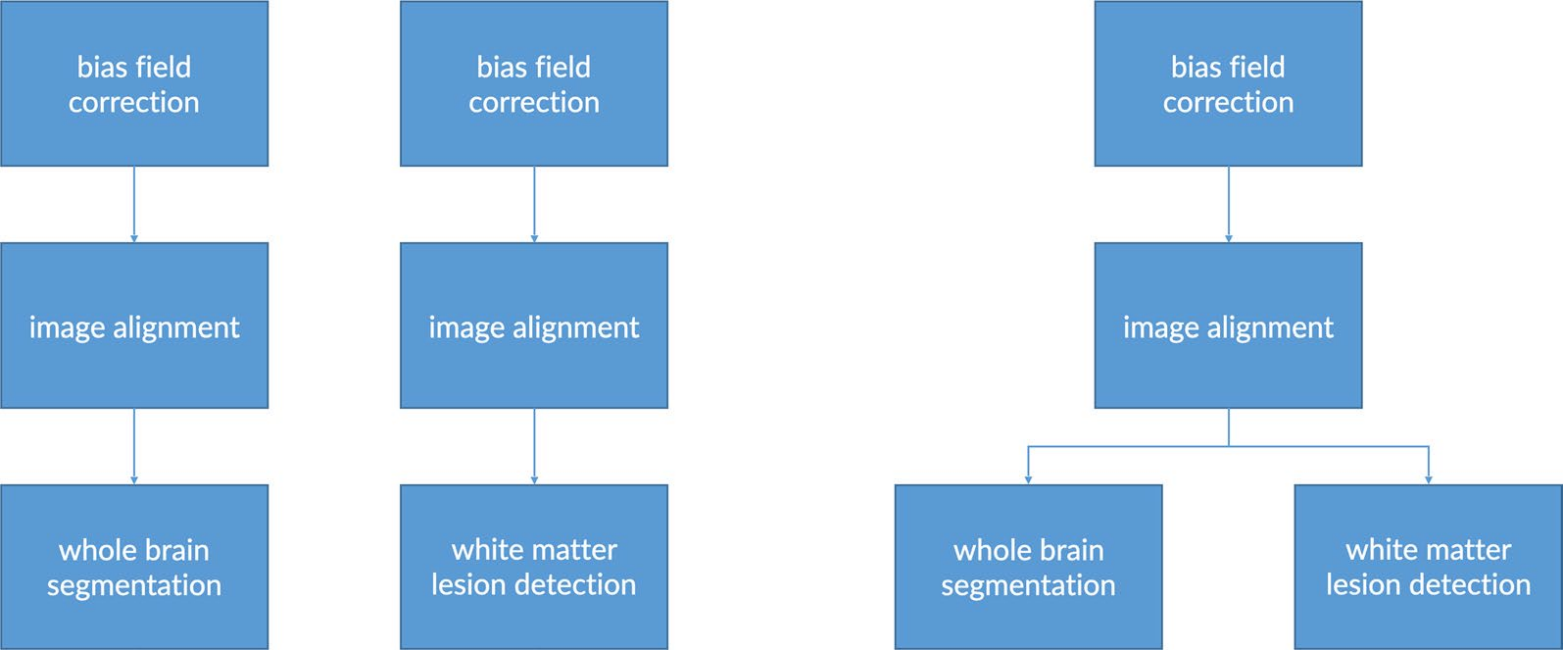
Illustration of the principle of using a directed acyclic graph (DAG) to efficiently pool common subtasks. Left panel shows a conventional approach with separate pipelines for different tasks (whole brain segmentation and white matter lesion detection). Right panel shows how using a DAG facilitates a more efficient approach that can accomplish the same end result by pooling common subtasks

Once the platform knows which pipelines should run for a specific examination, it constructs a worklist of all subtasks in the form of a directed acyclic graph (DAG). This DAG specifies which subtasks have to be finished before others can be started (Fig. 2). A priority task scheduler oversees all these DAGs and executes the subtasks, if possible, in parallel, and in an efficient order based on required resources, duration, workload and priority. Subtasks that require considerable computational resources but are not time critical may, for example, be scheduled for overnight processing.

### Algorithm versioning: a crucial component of responsible AI deployment

An additional benefit of using containerized applications is easy version control. Recent and older versions of the same container can be stored in a local registry and pulled from there when needed. This is a capability that is nearly impossible to achieve for point solutions. Versioning will prove to be a crucial component of responsible AI deployment. Because the essence of AI is learning from data, AI algorithms will continuously improve over time in contrast to deterministic CAD systems. This will make clinical judgement of longitudinal studies crucially dependent on being able to differentiate between evolution of disease and evolution of the neural network underlying the AI application. When we consider the example of a yearly follow-up study, a radiologist will want to see both the results of the current version of the AI algorithm, as well as the results of the algorithm version that ran on last year's data. An important advantage of a VNAI is that such a framework enables keeping older versions of a specific algorithm. That is, methods do not need to be replaced by updated versions, but newer versions become available alongside existing one(s). This, however, does not mean that all versions will be run in the usual workflow. It means that longitudinal studies can for example be performed with a constant, non-changing version of the method, if necessary. Changes to a method can be described in a change log. Such a setup can also reveal changes in output due to changes in the algorithm itself versus changes in a patients’ disease state. Thus, the IMAGR framework makes versioning more transparent and flexible compared to the present situation.

## Presenting AI output to the radiologist

### Types of output

An AI algorithm deployed via IMAGR may yield several types of output. In the most straightforward case, the output consists of derived images, overlays or annotations that can be pushed to the PACS and made available to the reading radiologist via his or her standard PACS viewer. As discussed above, the system is vendor neutral by design and can work with any PACS system that adheres to DICOM and HL7 standards. The output may also be a priority score or other metric that can be used to (de)prioritize examinations, thus influencing the order in which they are read. PACS vendors are starting to offer application programming interfaces (APIs) to support such worklist-influencing AI output. In the particular case of the Sectra PACS (Sectra AB, Linköping, Sweden) running at the UMCU, the API provides both a sorting parameter and a string variable to briefly describe why an examination received a certain score. In cases of more complex output, visualization in a dedicated viewer may be required.

### Visualization of results

Most standard PACS viewers support visualizing AI algorithm output as images, PDF reports or DICOM Structured Reporting (SR) objects. For AI algorithms that produce more complex results IMAGR has the option to add algorithm-specific graphical user interfaces (GUIs) to its portal website. For example, an algorithm may produce interactive annotations on 2D or 3D images, interactive graphs and tabular data, or plot a patient's individual results onto a normal distribution of a reference population or present a form that requires some manual interaction to select results.

**Fig. 3:**
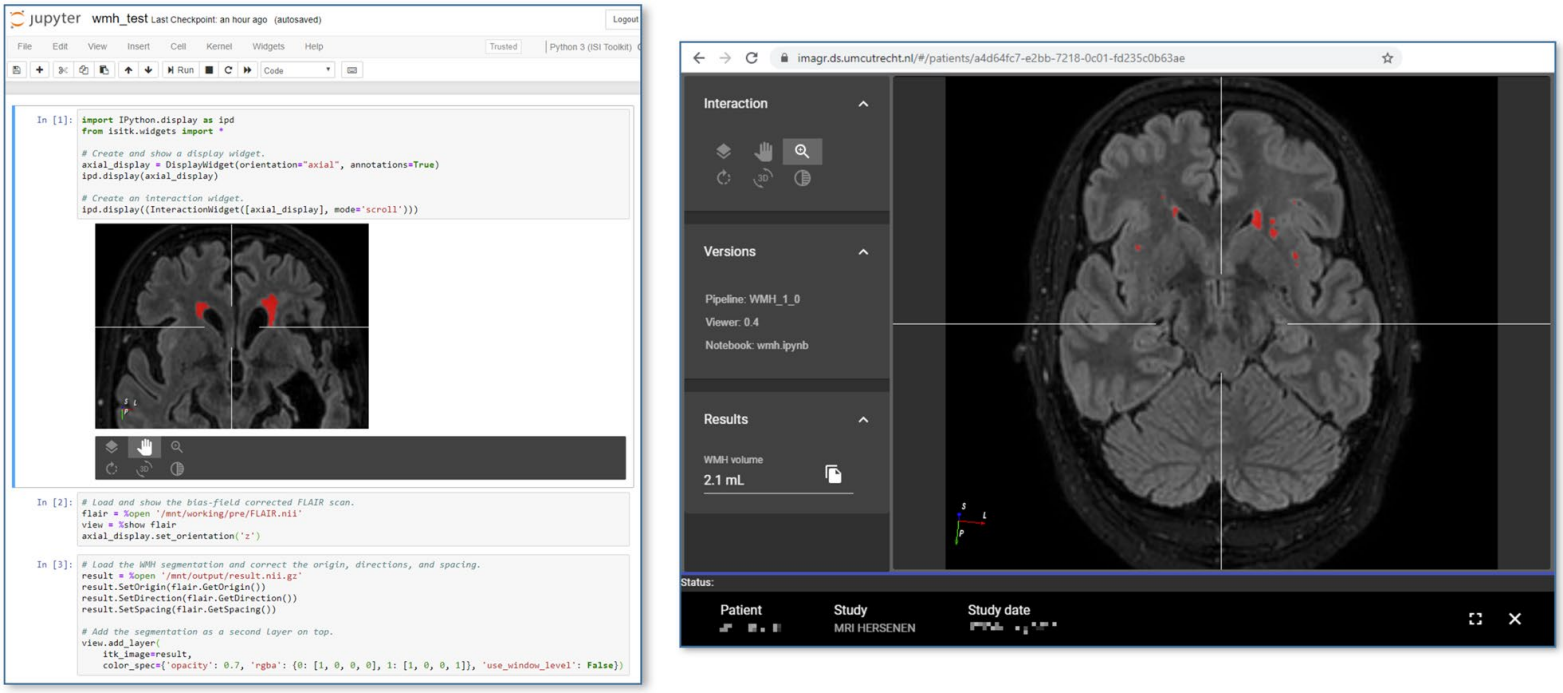
Example of a Jupyter Notebook with widgets for highlighting white matter hyperintensities in the brain. Left panel shows the source code of several widgets underlying the visualization in the graphical user interface (GUI) as displayed in the right panel. GUI elements consist of visualizing the acquired image including the image overlay, a control panel for manipulation the image stack (scrolling, panning, rotation and zooming), an information panel showing the pipeline version number as well as the underlying Jupyter Notebook and a results panel

Like the algorithms themselves, their GUIs can be split up in smaller building blocks or widgets. Widgets can be thought of as components for displaying 2D or 3D images, tool buttons for interaction, tables and graphs. By providing a set of standard widgets for algorithms to use, IMAGR makes the visualization of algorithm-specific GUIs uniform and user friendly. Widgets are programmed using the Jupyter Notebook environment [7], which is widely used by many researchers and AI developers. IMAGR uses a custom set of interactive Jupyter widgets that can be rendered in a standard Jupyter Notebook environment, but also in a GUI that integrates with the IMAGR portal website (Fig. 3).

### Workflow integration

By making AI results available on PACS workstations, whether as images pushed to the PACS or via a dedicated GUI, an important part of workflow integration has been realized. Results are available at the point of care, without the radiologist having to move to a different workstation or to start dedicated software. Another important part of integration is synchronization. IMAGR follows patient changes in the PACS and automatically shows all relevant AI output for the currently read study of the currently selected patient. In addition, a dashboard like website allows radiologists to search for patients and obtain information regarding the algorithms that are being run or have finished running on a patient's examinations. Furthermore, IMAGR can automatically alert the radiologist to AI pipelines that are waiting for user input. Examples are AI algorithms that require expert radiologist or technologist validation or selection of an intermediate result before finalizing its output. Supporting such semi-automatic AI pipelines via the above-described DAGs is an important requirement that sets a VNAI apart from standard scripted image post-processing services.

An important question that arises in this context is how radiologists should prioritize algorithm output in case multiple algorithms are executed or when similar algorithms provide conflicting outputs. This is not a problem that can or should be solved by the infrastructure technology itself. It underscores the principle that the radiologist remains responsible and ultimately decides what findings and interpretations to incorporate in the final report. Nevertheless, the AI infrastructure as described in the blueprint can aid in coming to a decision, by making it easy to run multiple algorithms—and even versions of algorithms—at the same time, while reading a case. Depending on the subject, the radiologist can then for example describe both results in the rapport or pick one of the two results as the basis for further evaluation. In addition, the ability to have dedicated viewing containers per algorithm may help to decide which algorithm is more likely to be correct. Even if the inner workings of the underlying neural networks are unknown, being able to inspect a resulting segmentation or activation heatmap, can often reveal whether results make sense.

Similar to PACS access, access to the IMAGR portal website is tied to corporate credentials by using the Lightweight Directory Access Protocol (LDAP) authentication. Using existing IT infrastructure and procedures guarantees that only authorized employees have access to the data and information in the system, and that access is revoked when people leave the institution.

### User interaction and valorization

Although for some AI applications presenting output as images sent to the PACS might be sufficient, images with burned-in overlays and PDF reports only allow for static content, and DICOM Structured Reporting (SR) objects—which can be used to store additional AI output—are often not rendered at all, or in a format that is not user-friendly. Interactive results are not only more informative, but also provide a means for a reader to give feedback. A VNAI that is able to record radiologist interaction and feedback on AI results in a structured manner provides enormous potential for valorization, not in the least because AI output is likely more structured compared to the generally free-text format of radiology reports, thereby reducing the current lack of structured data over time. User feedback is extremely valuable as additional training data to improve the underlying AI algorithm. In addition, a VNAI infrastructure that is able to quantitatively record the radiologist's interaction with an algorithm can facilitate collection of the much-needed evidence to demonstrate the economic impact of AI. Whether this results in fine-tuning an algorithm for a specific population or improvement in general, an arrangement that makes the intellectual effort of radiologists available to AI vendors can be mutually beneficial, while at same time improving patient care. Lastly a VNAI that is able to quantify user interaction lays the foundation for continuous quality monitoring and helps safeguard the patient against algorithmic errors.

Similar to visualizing results, GUIs that allow for user feedback can be defined using standardized set of Jupyter widgets to make the interaction as uniform and user-friendly as possible using a system with Jira-like tickets.

### Deploying multiple algorithms

To grow the VNAI, it is essential that AI vendors can easily hook into the infrastructure. That means the requirements for containerization should be heavily standardized, preferably through a global standard. Furthermore, the VNAI can function as an overarching umbrella structure that can support one or more AI-marketplaces, giving hospitals broader access to algorithms. In this way, the VNAI can drastically shorten the route from FDA/EMA-approved vendor product to actually clinical deployment, while at the same time vastly increasing the library of available products. Lastly, as mentioned earlier, because of the uniform interface, IMAGR allows the synergistic combining of algorithms at the user level, as mentioned earlier.

### Deployment variants

Institutions may decide to run a VNAI completely on-premise, providing the infrastructure for all the components. Nevertheless, we expect that a popular variant will be to outsource the GPU-heavy parts of the infrastructure to a cloud-provider because of the modular make-up of the infrastructure. The local hospital still controls what gets sent to the GPU-cluster and still determines what happens with the AI output—thereby maintaining patient confidentiality and control over the data as well as radiologist-feedback-valorization. Anonymization and pseudonymization are standard building blocks in IMAGR for all pipelines and should anonymized data leave the hospital, upon return of the results of an AI pipeline, the data can be reidentified behind the hospital firewall in accordance with the General Data Protection Regulation (GDPR) stipulations.

## Scientific perspective

Although the description above is mainly focused on a clinical scenario for using AI, IMAGR was designed from the onset with the realization in mind that AI requires a close collaboration between physicians and data scientists. Therefore, IMAGR not only supports a clinical user role, but also a scientific user role (Table 1).

**Table 1:** Differences between clinical and scientific user roles of VNAI

Clinical role	Scientific role
Pipeline trigger is per-patient HL7 notification or per-patient push to VNAI	Pipeline can be triggered to run on a specific set of data or specific data storage
Input is PACS	Input can be PACS or any other DICOM store; or even non-DICOM bulk storage
Pipelines mostly run immediately	Pipelines can be scheduled to off-hours
Portal API accessed at individual patient level	Portal API can be accessed also at pipeline level

This distinction is not only relevant to academic hospitals. It is likely that regulatory bodies may require local validation of certain types of algorithms on independent, validated datasets containing images from the local population or acquired using local scanning techniques. This type of per-hospital or per-local population validation will make use of the scientific user role and is applicable to both academic and non-academic radiology practices. Another example where AI can be useful is automated quality control, where the VNAI is tasked to analyse a random subset of—or all—studies acquired by individual scanners to find quality outliers based on pre-specified criteria, detect quality drift or perform general quality assurance tasks. With regard to quality control of algorithms themselves it is important to note that procedures for quality control are not different from those of ‘regular’ commercial or in-house developed applications. The exact quality criteria to be applied differ per application. If an AI algorithm was released that needs to be fixed or withdrawn, then the centralized design of the VNAI framework provides the means to do so and inform its users.

## Governance, quality control and ethics

### Governance

Algorithm deployment and usage on a VNAI provides unique advantages over point solutions. At the same time, it also requires a clear governance structure to ensure responsible use. Vendor-neutral AI deployment is a process that ultimately needs to be overseen by a multidisciplinary team of experts consisting of clinicians, algorithm developers, data scientists and hospital IT specialists. At UMC Utrecht such a team decides which algorithms to deploy and exactly how this is done. Specifically, a dedicated team is appointed for each algorithm because we believe that bringing together in-dept knowledge of the specific algorithm, in-depth clinical domain knowledge as well as understanding of the IT aspects of deployment, results in the most responsible timing and method of deployment and offers the best opportunity for monitoring the implementation. Ethics and legal experts as well as patients can also be involved on the implementation team, depending on the kind of algorithm and intended use cases.

### Quality control

Proper quality control is essential for AI algorithms that are made available to end users. Currently, there are no agreed upon specific guidelines or best practices regarding deployment of AI algorithms via a VNAI. When discussing quality control, a separation needs to be made between the individual algorithms and the VNAI on which they are deployed.

While it is likely that for example the visualization components of the VNAI itself will be regarded a 'medical device' in the terms of the EU Medical Device Regulation law [8], we believe that when deciding on quality control of the algorithms, it is crucial to take into account the nature of the each specific algorithm and the way it will be deployed and used. As the VNAI will become an important component in the radiology workflow, it is clear that its building blocks should adhere to existing software quality assurance procedures during the various phases of development (planning, requirements, architecture, detailed design, implementation, unit testing, integration testing, system, and acceptance testing) and ultimately deployment and maintenance.

End users will undergo training both in using the VNAI and in understanding the output of AI algorithms. This also demands that radiologists become ‘AI-capable’, i.e. that they understand the strengths and weaknesses of algorithms running on a VNAI. In other words, similar to how radiologists and technicians are aware of current imaging limitations and artefacts that may interfere with reporting, they should become aware of AI-artefacts that can be present in the results of algorithms. Knowing when to trust the output of an algorithm and when to ignore it and being able to recognize pitfalls is important for quality control and will likely become a standard part of training and education. Although there is presently no consensus on a comprehensive set of relevant metrics by which to judge the output of AI algorithms, devising such criteria should be a joint priority of AI researchers and clinicians seeking to apply algorithms in clinical practice.

Furthermore, it is important to consider the scenario where input from another radiologist or non-medical expert is required, such as in a multidisciplinary conference. IMAGR supports asynchronous execution and can halt a pipeline for a required manual step. Pipeline progress is shown in a patient's list of AI results waiting for human action (either "read" when finished, or "do manual task" when waiting for manual task). Depending on the exact implementation, the pipeline may in addition send out a notification that action is required. In case of any doubts, another radiologist could simply rerun the pipeline by sending the examination to IMAGR and then perform any required manual step(s) according to his/her own expertise.

Finally, it is important to consider security aspects. The IMAGR servers—and thus the algorithm containers—run on the same firewalled network as the PACS servers. Having IMAGR store AI-derived output in the PACS therefore does not increase or decrease the security of the PACS. Also, the containers themselves run with minimal user rights making them unattractive as possible origin point for an attack, if, for instance, the AI vendors software was compromised.

### Ethical aspects

AI deployment raises a number of ethical considerations. For all aspects of development and deployment of IMAGR we strive to adhere to the principles of responsible use of AI as laid down by the EU [9].

**Fig. 4:**
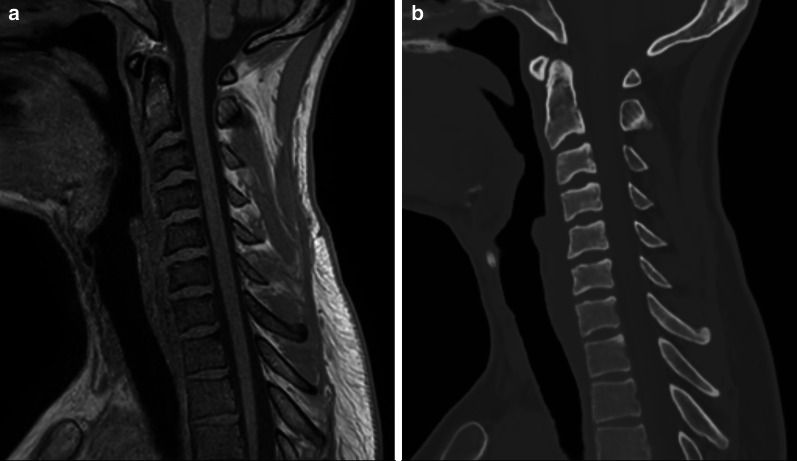
The CE-marked BoneMRI software product [16] (BoneMRI V1.1, MRIguidance BV, Utrecht, The Netherlands) has been provided as a self-contained application connected to the local PACS through the IMAGR platform. The figure shows T1-weighted MRI (left) and reconstructed BoneMRI (right) images of the cervical spine of a healthy volunteer. The 3D BoneMRI image is reconstructed from the T1wMRI images using the BoneMRI software and pushed back into PACS

**Fig. 5:**
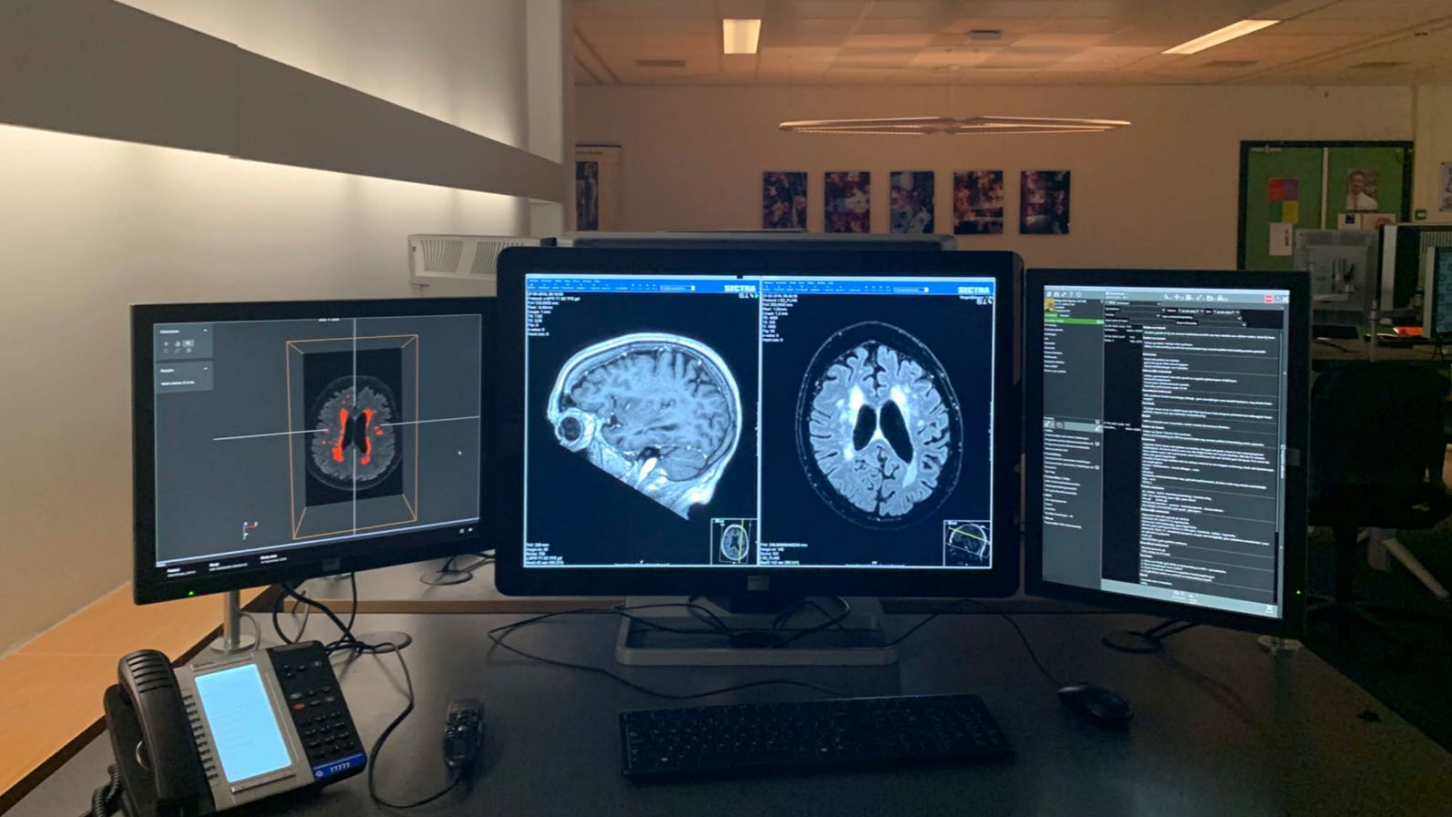
Example of VNAI integration in clinical workflow. The PACS system (middle monitor) and hospital information system (right monitor) running side-by-side. The left monitor shows the output of a white matter hyperintensity quantification algorithm [17]. The user can scroll through the stack of images and correlate the algorithms output with the source images in the PACS

While deployment of algorithms with regulatory approval is relatively straightforward (Fig. 4), this may be different for research algorithms that lack FDA or EMA approval. Running research algorithms in the context of clinical trials demand either a waiver of the Medical Ethics Committee or pre-approval and patient-informed consent. A third situation to consider is the use of AI algorithms to create local normal values. An example is using AI to calculate detailed, local reference ranges for various parameters such as the size of anatomical structures, body composition, coronary calcium, white matter hyperintensities in the brain (Fig. 5). As long as algorithms are properly validated and processing occurs anonymously, we believe this should be considered part of routine clinical care, analogous to keeping track of clinical reference values, outcomes or distributions without using AI. Another question concerns responsibility for errors. Similar to currently deployed CAD algorithms, the clinician remains responsible for the final conclusions of the image interpretation and report, including the findings based on information generated by AI algorithms. A detailed discussion of the legal responsibilities of algorithm creators versus radiologists is beyond the scope of this paper, and we refer the reader to article by Nicholson-Price for more information [10]. As discussed in the section on governance above, we believe that digital medicine should be a co-production [11] that involves both medical and imaging experts and should take into account patient and societal perspectives in order to maximize trust and to guarantee transparency.

## Future perspectives

### Radiology use cases other than examination post-processing

Hardware virtualization and software containerization are techniques that can be beneficial in any environment where computing resources should be available on demand. In the radiology department, this also applies to image reconstruction. Reconstructing MR or CT acquisitions requires significant computational power, especially when it comes to using modern iterative reconstruction algorithms, but most of the time the dedicated hardware is sitting idle. Sharing those resources through a VNAI could benefit both reconstruction (a large computation pool can be made available when an acquisition needs to be reconstructed) and image analysis (hardware that is usually assigned to reconstruction can be assigned to analysis when scanners are idle). Extending the VNAI infrastructure to the scanner console also facilitates for example AI-accelerated MRI-acquisition or reconstruction [12].

Furthermore, a VNAI can be used for automated quality control of acquisition hardware. This can be done in a traditional way, i.e. by automatically running diagnostic tests when phantoms are scanned, but AI may also be used to continuously monitor image quality by (randomly) analysing acquired data.

Finally, having one centralized system for acquisition post processing also allows for aggregating information from multiple sources. For imaging data this could mean that scans from different modalities or follow-up examinations could be automatically registered, overlaid and compared.

### Imaging biomarker development

The VNAI can also serve as a platform to both develop and validate new imaging biomarkers based on AI algorithms, as well as more conventional mathematical models. Developing new biomarkers is a multidisciplinary research task which requires input from physicians, engineers, data scientists, statisticians, etc. Being able to process data in bulk and aggregate data from multiple image processing pipelines, the VNAI may aid in biomarker development, testing, validation and implementation. Many potential imaging biomarkers identified by AI algorithms do not make it beyond the discovery or proof-of-concept stage, in part because it is hard to scale beyond the local dataset and include data from multiple centres. The use of a VNAI may facilitate the next stages in imaging biomarker validation programs, providing resources (hardware, software, access to data) to perform repeatability and proof-of-principle studies. This will have the largest impact in bridging the translational gap, i.e. bringing biomarkers and AI algorithms from a single centre to multiple centres [13, 14]. In the later stages of imaging biomarker development, the VNAI can support clinical trials and, ultimately, the same VNAI can be used to adopt novel AI-based imaging biomarkers in clinical routine [15]. As with AI output it is important to realize that human input remains necessary for selecting sensible biomarkers in the context of a specific disease and for all stages of the imaging biomarker development.

### Beyond imaging data

Although initially deployed in the setting of a radiology department, our VNAI platform is designed to accommodate a broad variety of medical data formats. This makes the platform ideally suited to function as the central ‘AI-hub’ in the hospital. In addition to the standard DICOM format, IMAGR is capable of working with almost any data format such as data from laboratory information systems, electronic health records and data from genomic analyses. For example, IMAGR can directly connect with digital pathology data—which currently does not conform to the DICOM standard—in the Sectra Pathology PACS. The obvious advantage of this approach is a much lower barrier to development and deployment of AI algorithms that take into account data from multiple different modalities or domains. We expect that this will lead to tangible benefits for patients over time.

## Conclusions

AI provides tremendous opportunities for improving patient care, but at present there is little evidence of real-world uptake. An important barrier is the lack of well-designed, vendor-neutral and future-proof infrastructures for deployment. Because current AI algorithms are very narrow in scope, it is expected that a typical hospital will deploy many algorithms concurrently. Managing stand-alone point solutions for all of these algorithms will be unmanageable. A solution to this problem is a dedicated platform for deployment of AI. We have provided an overview of the high-level design and implementation considerations of such a system—which is up and running at UMC Utrecht—that can be used clinically as well as for research and development. Close collaboration between radiologists, data scientists, software developers and experts in hospital IT as well as involvement of patients is crucial in order to successfully bring AI to the clinic.

## Abbreviations

AI: Artificial intelligence
API: Application programming interfaces
DAG: Directed acyclic graph
DICOM: Digital Imaging and Communications in Medicine
GPU: Graphics processing units
HIS: Hospital information system
HL7: Health level 7
LDAP: Lightweight Directory Access Protocol
PACS: Picture archiving and communication system
SR: Structured reporting
VNAI: Vendor-neutral AI deployment infrastructure
